# Changes in the size and electrophoretic mobility of HDL subpopulation particles in chronic kidney disease

**DOI:** 10.1007/s40620-022-01412-y

**Published:** 2022-08-09

**Authors:** Anna Gliwińska, Agnieszka Ćwiklińska, Monika Czaplińska, Ewa Wieczorek-Breitzke, Barbara Kortas-Stempak, Agnieszka Kuchta, Alicja Dębska-Ślizień, Ewa Król, Maciej Jankowski

**Affiliations:** 1 Department of Clinical Chemistry, Medical University of Gdańsk, Dębinki 7, 80-211 Gdańsk, Poland; 2 Department of Nephrology, Transplantology and Internal Diseases, Medical University of Gdansk, Dębinki 7, 80-211 Gdańsk, Poland

**Keywords:** Chronic kidney disease, High-density lipoprotein, HDL subpopulation, 2D-PAGGE

## Abstract

**Background:**

High-density lipoprotein (HDL) is a heterogeneous group of particles with anti-atherogenic properties whose metabolism is alterated in chronic kidney disease (CKD). The aim of this study was to evaluate the particle size and mobility of HDL subpopulations in non-dialysis CKD patients.

**Methods:**

The study involved 42 non-dialysis CKD patients (stages 3a–4) and 18 control subjects. HDL was separated by non-denaturing two-dimensional polyacrylamide gradient gel electrophoresis (2D-PAGGE) and eight HDL subpopulations; preβ1, preβ2a-c, and α1-4 were distinguished. The size and electrophoretic mobility of HDL subpopulation particles were compared between the groups, and a regression analysis was conducted.

**Results:**

In CKD patients, the mean sizes of α-HDL and preβ2-HDL particles were significantly lower compared to the control group (8.42 ± 0.32 nm vs. 8.64 ± 0.26 nm, *p* = 0.014; 11.45 ± 0.51 vs. 12.34 ± 0.78 nm, *p* = 0.003, respectively). The electrophoretic mobility of preβ2-HDL relative to α-HDL was significantly higher in CKD patients compared to the control group (Rf 0.65 ± 0.06 vs. 0.53 ± 0.10, *p* = 0.002). The size and mobility of HDL subpopulations correlated with eGFR values (*p* < 0.01). These relationships remained statistically significant after adjusting for age, gender, statin treatment, apolipoprotein AI, total cholesterol, and triglyceride levels.

**Discussion:**

CKD affects the size and mobility of HDL particles, which can be related to HDL dysfunction. The magnitude of HDL size and mobility changes depended on CKD stage and differed for individual HDL subpopulations, which indicates that some stages of HDL metabolism may be more affected by the presence of chronic kidney disease.

**Graphical abstract:**

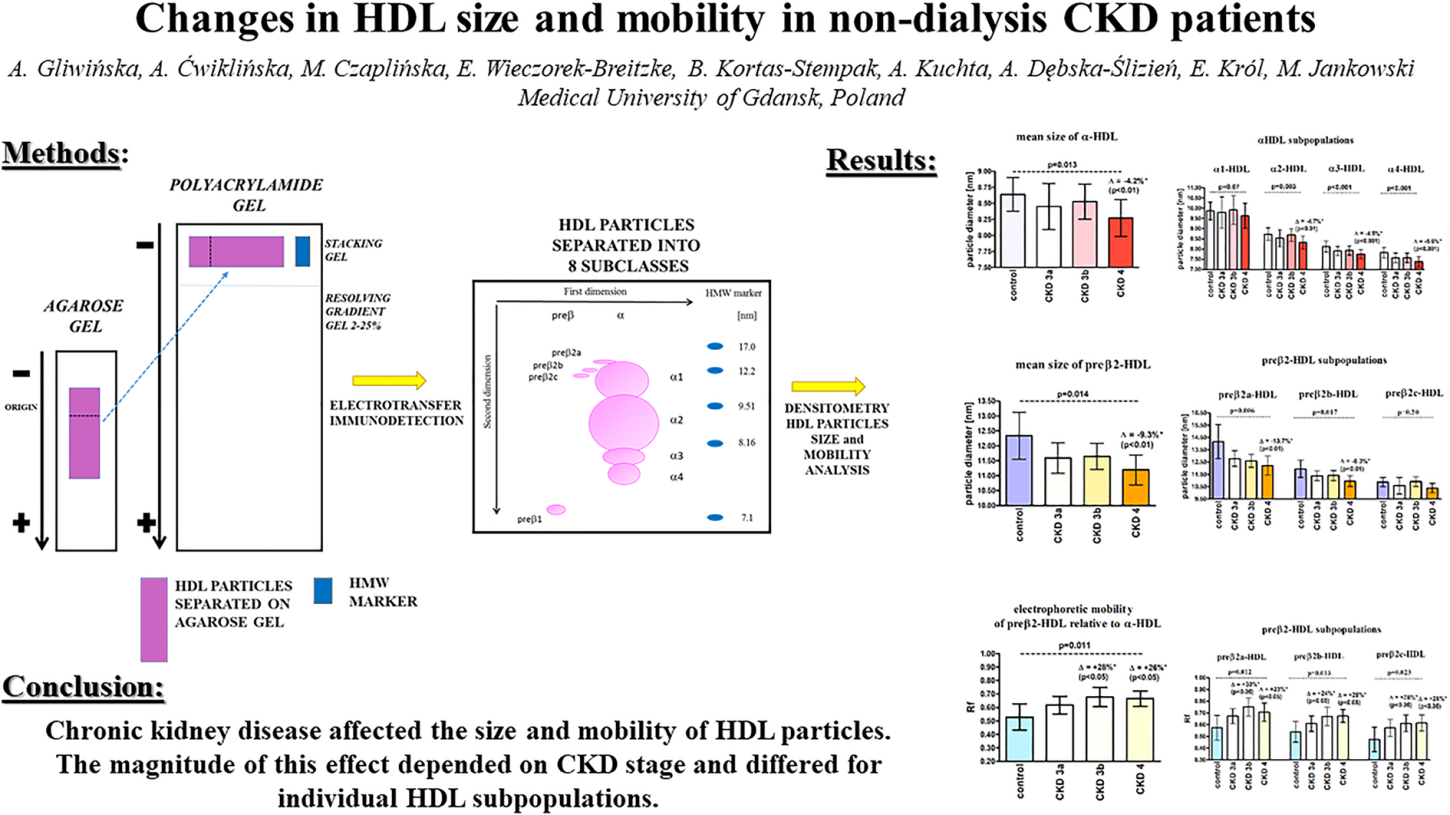

## Introduction

Chronic kidney disease (CKD) is associated with a high risk of atherosclerosis and cardiovascular disease (CVD), which is the leading cause of morbidity and mortality in CKD patients [1]. One of the main causes of CVD development in CKD is a significant disturbance of lipid metabolism, which is related to quantitative and qualitative changes in lipoproteins, including high-density lipoprotein (HDL) [2].

Many studies have shown an inverse relationship between HDL cholesterol (HDL-C) levels and cardiovascular risk, but pharmacological interventions to increase HDL-C failed to reduce cardiovascular endpoints [3]. Moreover, it was observed that HDL became dysfunctional in several diseases, including CKD [4]. Therefore, nowadays, the quality and function of HDL are considered more important for the atheroprotective activity of HDL than the total quantity assessed by the HDL-C level [5].

HDL particles can be divided into subpopulations, and their mutual transformation is a continuous process that determines their functions [6]. The basic structural and functional apolipoprotein of HDL is apolipoprotein AI (apoAI) [7]. By interacting with ATP binding cassette transporter A1 (ABCA1), apoAI uptakes cholesterol and phospholipids from cells to form the smallest HDL particles with preβ1 electrophoretic mobility. The newly formed particles undergo further transformations in plasma in the presence of enzymes and lipid transfer proteins, such as lecithin:cholesterol acyltransferase (LCAT) and cholesteryl ester transfer protein (CETP). The ongoing processes affect the physicochemical properties of HDL particles, the effect of which is an increase of their size, the change of their shape from discoidal to spherical, and the change of their mobility from preβ to α. In the final stage of reverse cholesterol transport (RCT), large HDL particles transfer cholesterol to the liver and degrade into small and very small particles that can be excreted by the kidneys or recycled into HDL as part of its remodeling in plasma [8].

Numerous methods based on differences in particle density, size, mobility, and composition have been used to separate HDL subpopulations [6]. Ultracentrifugation allows HDL to be divided according to density into HDL-2 and HDL-3. Gradient gel electrophoresis (GGE) allows HDL to be separated into two HDL-2 subclasses (HDL-2a and HDL-2b) and three HDL-3 subclasses (HDL-3a, HDL-3b, and HDL-3c) according to their sizes. Nuclear magnetic resonance (NMR) allows the three HDL subclasses (large, medium, and small) to be separated and the HDL particle count to be quantified. A high-resolution method that allows HDL particles to be separated according to surface charges and sizes is two-dimensional non-denaturing polyacrylamide gradient gel electrophoresis (2D-PAGGE). This method enables differentiation of HDL subpopulations with preβ and α mobility, namely preβ1-HDL, preβ2-HDL (preβ2a-HDL, preβ2b-HDL, and preβ2c-HDL), and α-HDL (α1-HDL, α2-HDL, α3-HDL, and α4-HDL) subpopulations [6].

Despite the link between HDL quality and CVD development, most research on HDL disturbances in CKD has focused on changes in the quantity or function of the entire HDL fraction and on advanced stages of renal impairment. However, little is known about modifications of HDL subpopulations developing along with CKD progression that can affect HDL atheroprotective properties. Therefore, the aim of our study was to evaluate HDL subpopulation particle profiles in non-dialysis CKD patients using the 2D-PAGGE method.

## Methods

### Subjects

The study included 60 adult subjects: 42 patients with stage 3a to 4 CKD, treated conservatively at the University Clinical Center in Gdańsk (Poland), and 18 non-CKD subjects, who comprised the control group. The exclusion criteria were diseases that were related to lipoprotein disturbances or that substantially affected metabolic balance, namely diabetes, liver diseases, active malignancy, acute diseases within three months before the study, and nephrotic proteinuria. Moreover, the exclusion criteria included treatment with immunosuppressive agents, including steroids, heparin treatment, and hypolipidemic treatment, except for low doses of statins. The characteristics of the study groups are presented in Table 1.

**Table 1:** Characteristics of study groups

	Controls	CKD patients				*p*
		All	Stage 3a	Stage 3b	Stage 4	
*n*	18	42	16	13	13	–
Male/female	10/8	23/19	10/6	6/7	7/6	0.85^a^
Age	61 ± 7	66 ± 11	67 ± 11	67 ± 8	64 ± 14	0.10^b^
BMI	27 ± 4	27 ± 5	27 ± 5	30 ± 5	25 ± 5	0.12^b^
Statin treatment	6 (33%)	20 (48%)	5 (31%)	7 (54%)	8 (61%)	0.26^a^
Creatinine [mg/dl]	0.87 ± 0.19	1.75 ± 0.62	1.25 ± 0.16	1.60 ± 0.28	2.50 ± 0.43	< 0.001^b^
eGFR CKD-EPI [ml/min/1.73m^2^]	84 ± 14	39 ± 13	53 ± 5	39 ± 5	23 ± 4	< 0.001^b^
TC [mg/dl]	199 ± 34	208 ± 46	202 ± 47	205 ± 56	220 ± 33	0.60^b^
LDL-C [mg/dl]	118 ± 34	133 ± 42	126 ± 42	133 ± 54	142 ± 31	0.42^b^
TG [mg/dl]	107 [62–175]	116 [90–151]	113 [76–145]	136 [94–157]	108 [93–142]	0.81^c^
HDL-C [mg/dl]	55 ± 12	51 ± 12	52 ± 13	47 ± 9	53 ± 13	0.25^b^
apoAI [mg/dl]	189 ± 23	164 ± 26	166 ± 25	157 ± 26	169 ± 28	0.006^b^

Data is presented as mean ± SD or median [IQR]. *p* value—comparison of control and CKD stage 3a, 3b and 4 groups

*BMI* body mass index; *eGFR CKD-EPI* estimated glomerular filtration rate according to Chronic Kidney Disease Epidemiology Collaboration formula; *TC* total cholesterol; *LDL-C* low-density lipoprotein cholesterol; *TG* triglyceride; *HDL-C* high-density lipoprotein cholesterol; *apoAI* apolipoprotein AI; *SD* standard deviation; *IQR* interquartile range

^a^*χ*^2^ test

^b^ANOVA test

^c^Kruskal–Wallis test

### Biochemical analyses

Blood was collected after an overnight fast to obtain serum. Creatinine was measured using the enzymatic method (Abbott Diagnostics Inc., Santa Clara, CA, United States). The estimated glomerular filtration rate (eGFR) was calculated according to the CKD Epidemiology Collaboration (CKD-EPI) formula. Depending on the eGFR value, the patients were classified into CKD stages 3a (45–59 ml/min/1.73 m^2^), 3b (30–44 ml/min/1.73 m^2^), and 4 (15–29 ml/min/1.73 m^2^) [9].

HDL was isolated from the serum using the heparin–manganese chloride precipitation method. Total cholesterol (TC), HDL-C, and triglyceride (TG) levels were determined using the enzymatic method (Pointe-Scientific, Warsaw, Poland). Low-density lipoprotein cholesterol (LDL-C) was calculated using the Friedewald formula. ApoAI was determined using the immunonephelometric method (Siemens Healthcare Diagnostics, Erlangen, Germany).

### 2D-PAGGE electrophoresis

The isolated HDL fraction was dialyzed with 0.1 M Tris–HCl buffer pH 7.4 and subjected to 2D-PAGGE, as described previously [10], with some modifications. Briefly, in the first dimension, the HDL fraction (8 µg apoAI) was separated on agarose gel (0.75% w/v, 0,192 M Tris–glycine buffer, pH 8.5, 10 ºC). The agarose gel containing HDL particles was then transferred into a polyacrylamide gel. In the second dimension, the HDL was separated (2–25%, 160 V, 16 h, 10 ºC). A High Molecular Weight Native Marker Kit (HMW, GE Healthcare, United Kingdom) was run on each gel as a standard. After electrophoresis, the particles were electrotransferred onto PVDF membrane (4 ºC, 30 V, 26 h). Next, immunodetection with mouse anti-human apoAI antibodies (Monoclonal Anti-APOAI, Sigma-Aldrich, United States) and secondary polyclonal antibodies (Anti-Mouse IgG, Sigma-Aldrich, United States) labeled with alkaline phosphatase and using NBT/BCIP as chromogenic substrates was performed.

**Fig. 1:**
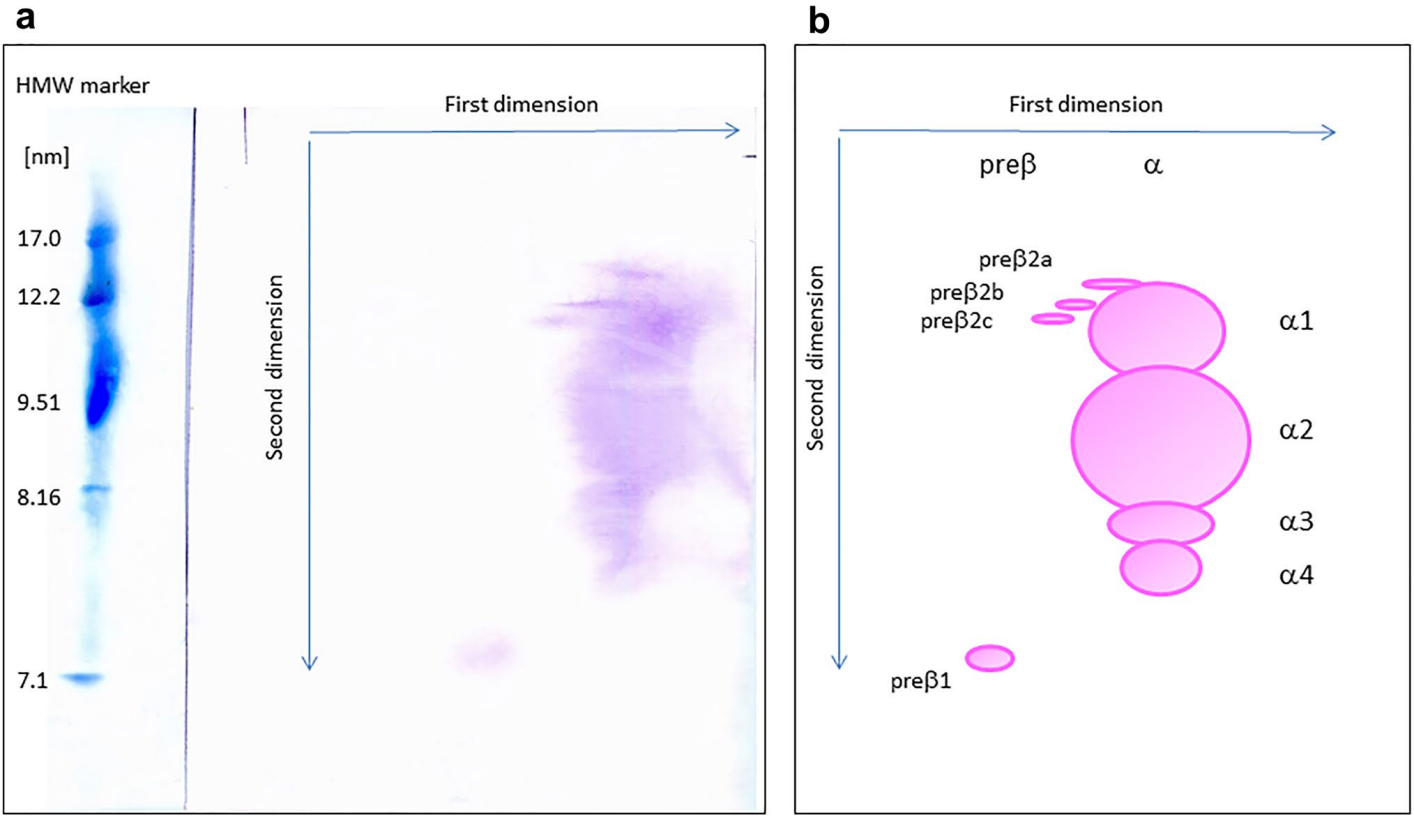
Separation of HDL subpopulations using two-dimensional non-denaturing polyacrylamide gradient gel electrophoresis (2D-PAGGE). **a** exemplary electropherogram of HDL subpopulations obtained for CKD subjects; **b** schematic diagram of HDL subpopulations

On the electropherograms, the presence of eight HDL subpopulations with different mobilities and sizes was distinguished. Namely, it was preβ1-HDL, three preβ2-HDL subpopulations (preβ2a, preβ2b, and preβ2c), and four α-HDL subpopulations (α1, α2, α3, and α4) (Fig. 1).

Densitometric analysis of the HDL subpopulations was performed using GelAnalyzer 2010 software.

The modal diameters of the HDL subpopulations were determined by comparing the mobility of the subpopulation peaks on the densitograms with the mobility of globular protein standards (HMW) electrophoresed on the same gel. The mean sizes of α-HDL and preβ2-HDL were calculated as the mean values of modal diameters obtained for individual subpopulations.

The relative electrophoretic mobility (Rf) for preβ2-HDL subpopulations was determined as the migration distance of individual preβ2-HDL subpopulation peak divided by the maximum of the migration distance for α-HDL in the 1st dimension of 2D-PAGGE. The mean of preβ2-HDL relative electrophoretic mobility was calculated as the mean value of the relative electrophoretic mobilities for individual preβ2-HDL subpopulations.

### Statistical analysis

The results were analyzed using the GraphPad Prism 4.0 and Statistica™ 13.3 programs. The categorical variables were expressed as numbers and percentages and analyzed using Pearson’s *χ*^2^ test. The normality of the continuous data distribution was assessed with the Shapiro–Wilk test, and the data were presented as means and standard deviations (SDs) or medians with interquartile ranges (IQRs), as appropriate. The differences between the two groups were assessed using a non-paired *t*-test. The differences between more than two groups were assessed using ANOVA with a Tukey post hoc test or a Kruskal–Wallis test, if appropriate. Univariate and multivariate stepwise linear regression analyses were performed to identify the associations between renal function assessed by eGFR CKD-EPI and HDL particle size and mobility. Logarithmic transformations were applied before regression analyses, when appropriate, to approach Gaussian distribution. Variables that showed significant correlations with HDL size in the univariate analysis or were considered a priori to have possible metabolic significance for HDL size distribution (age, gender, statin treatment, apoAI, TC, and TG level) were used as potential covariates in the multivariate regression analyses. The HDL-C level was not included, since it was closely correlated with both apoAI and TG levels. Values of *p* < 0.05 were considered statistically significant.

## Results

### Comparison of baseline characteristics and lipid parameters between CKD and non-CKD patients

The control and CKD groups did not differ significantly in terms of age, gender, BMI, or statin treatment. In addition, there were no significant differences in TC, LDL-C, TG, and HDL-C serum levels. The apoAI concentration was, on average, 13% lower in the CKD groups compared to the control group (Table 1).

### Analysis of HDL subpopulation sizes

**Fig. 2:**
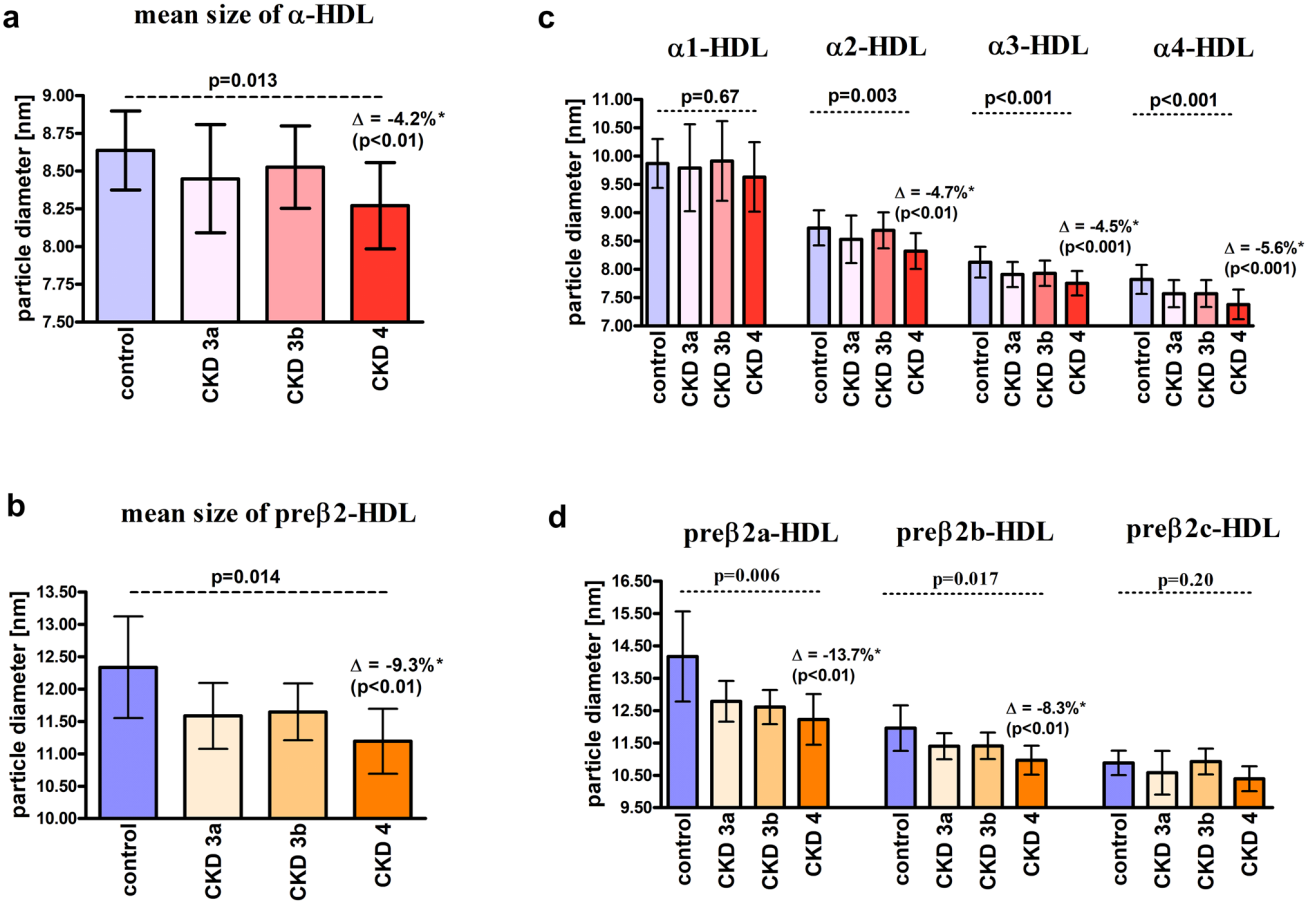
HDL particle sizes for the CKD patients and control group. **a** the mean size of α-HDL particles; **b** the mean size of preβ2-HDL particles; **c** the sizes of α-HDL subpopulations; **d** the sizes of preβ2-subpopulations. Data is presented as mean ± SD; *p*—ANOVA test, *vs. control group (with post hoc test *p* value in brackets)

For CKD patients, the mean sizes of α-HDL and preβ2-HDL particles were 8.42 ± 0.32 nm and 11.45 ± 0.51 nm, respectively, and they were significantly lower compared to the control group (8.64 ± 0.26 nm, *p* = 0.014 and 12.34 ± 0.78 nm, *p* = 0.003, respectively). There was no difference for preβ1-HDL particle diameter between the groups (6.75 ± 0.32 nm vs. 6.71 ± 0.31 nm, *p* = 0.77). The smallest mean dimensions of α-HDL and preβ2-HDL particles were observed for the patients with stage 4 CKD (Fig. 2a, b).

Regarding α-HDL subpopulations, we observed a decrease in HDL particle size in CKD patients for all subpopulations except for the largest α1-HDL particles and the greatest difference (− 5.6% for stage 4 CKD vs. the control) for the smallest α4-HDL particles (Fig. 2c). Conversely, a significant difference in particle size between the groups was observed for all preβ2-HDL subpopulations except for the smallest preβ2c-HDL particles. The greatest difference (− 13.7% for stage 4 CKD vs. the control) was observed for the largest preβ2a-HDL particles (Fig. 2d).

### Analysis of the relative electrophoretic mobility of HDL subpopulations

**Fig. 3:**
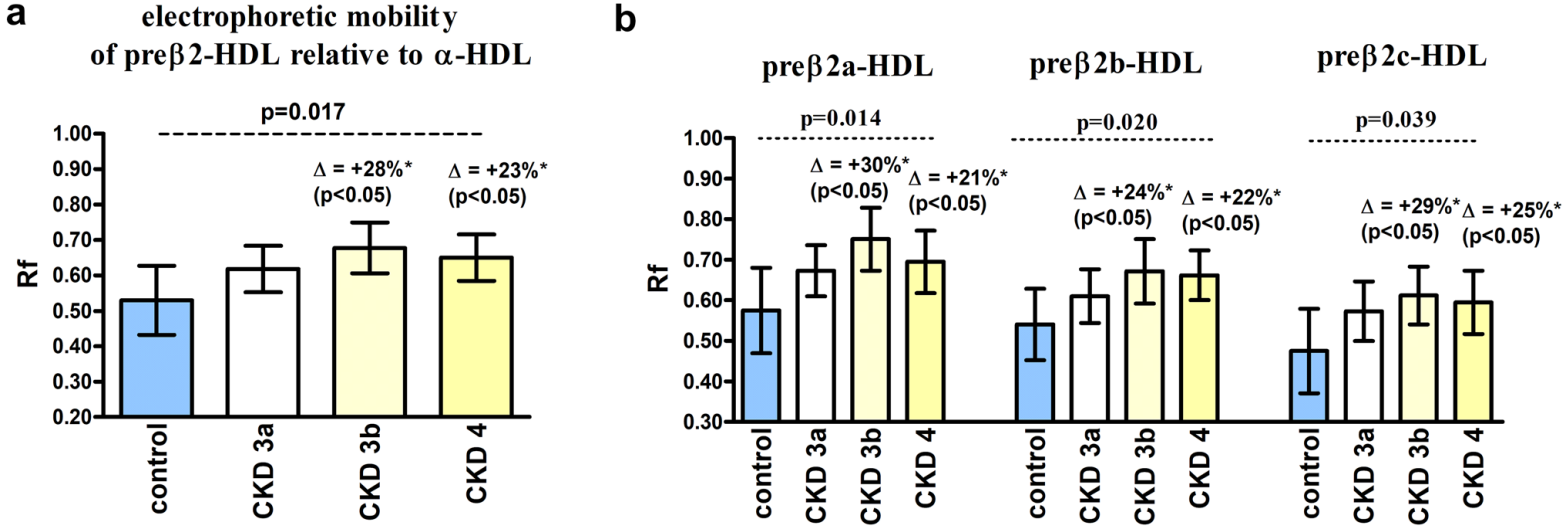
Mobility of preβ2-HDL particles relative to α-HDL for the CKD patients and control group. **a** The mean relative mobility of preβ2-HDL particles; **b** the relative mobility of preβ2-HDL subpopulations. Data is presented as mean ± SD; p—ANOVA test, *vs. control group (with post hoc test *p* value in brackets)

For CKD patients, the electrophoretic mobility of preβ2-HDL relative to α-HDL was significantly higher compared to the control group (Rf 0.65 ± 0.06 vs. 0.53 ± 0.10, *p* = 0.002). Significant differences in relative particle electrophoretic mobility between the control and CKD groups were observed for each preβ2-HDL subpopulation (Fig. 3).

### The relationship between glomerular filtration rate and the sizes, and electrophoretic mobility of HDL subpopulations

Univariable linear regression showed a statistically significant relationship between the glomerular filtration rate assessed with CKD-EPI [ml/min/1.73 m^2^] and the mean α-HDL sizes and the sizes of α-HDL subpopulations, except for α1-HDL. For HDL particles with preβ2-mobility, CKD-EPI correlated with the mean preβ2-HDL size and the sizes of preβ2a and 2b subpopulations but not with preβ2c size. For the relative electrophoretic mobility of preβ2-HDL, CKD-EPI correlated negatively with both the mean value and the mobilities for individual preβ2-HDL subpopulations. All these relationships remained statistically significant in the multivariable regression model after adjusting for age, gender, statin treatment, apoAI, TC, and TG levels (Table 2).

**Table 2:** Univariable and multivariable regression analysis between eGFR CKD-EPI [ml/min/1.73 m^2^], HDL particle size and relative electrophoretic mobility

	Univariate			Multivariate^a^		
	*β*	SE	*p*	*β*	SE	*p*
*HDL subpopulation particle size*						
Mean α-HDL [nm]	0.388	0.121	0.002	0.410	0.119	0.001
α-HDL subpopulations:						
α1-HDL [nm]	0.144	0.130	0.27	–	–	–
α2-HDL [nm]	0.333	0.124	0.009	0.377	0.119	0.002
α3-HDL [nm]	0.479	0.115	< 0.001	0.479	0.115	< 0.001
α4-HDL [nm]	0.523	0.112	< 0.001	0.693	0.141	< 0.001
Mean preβ2-HDL [nm]	0.510	0.179	0.009	0.678	0.189	0.002
preβ2-HDL subpopulations:						
preβ2a-HDL [nm]	0.525	0.177	0.007	0.462	0.162	0.009
preβ2b-HDL [nm]	0.523	0.178	0.007	0.546	0.156	0.001
preβ2c-HDL [nm]	0.291	0.199	0.157	–	–	–
*preβ2-HDL electrophoretic mobility relative to α-HDL*						
Mean for preβ2-HDL [Rf]	− 0.451	0.186	0.023	− 0.497	0.184	0.014
preβ2-HDL subpopulations:						
preβ2a-HDL [Rf]	− 0.413	0.189	0.040	− 0.457	0.190	0.026
preβ2b-HDL [Rf]	− 0.479	0.183	0.015	− 0.553	0.179	0.005
preβ2c-HDL [Rf]	− 0.422	0.189	0.036	− 0.424	0.188	0.035

^a^After adjustment for age, gender, statin treatment, apoAI, TC and TG level

## Discussion

In this study, we report changes in the size and mobility of HDL subpopulations in non-dialysis CKD patients. We found that the mean size of HDL particles with α- and preβ2-mobility was significantly lower and the relative preβ2-HDL mobility was significantly higher in CKD patients compared to the controls. Moreover, we found that the magnitude of these changes depended on the CKD stage and differed for individual HDL subpopulations.

Mean HDL size can be regarded as an integrative measure of the HDL particle profile, and a reduced mean HDL particle size typically relates to an increased CVD risk [11]. Watanabe et al., using GGE, found that Finnish subjects with familial low HDL had a decreased HDL particle size that correlated with increased carotid intima-media thickness [12]. Asztalos et al. found lower levels of large α1 particles and higher levels of smaller α3 particles in patients with CVD compared to the HDL-C-matched control group, as well as an inverse association of the level of large HDL with a risk of developing CVD [13]. However, the metabolic basis for the reduced HDL particle size in CVD is still unknown [14].

A decrease in the mean HDL particle size was also observed in CKD patients, but the results of the studies are inconsistent. The NMR study showed that patients with eGFR < 45 mL/min/1.73 m^2^ had a significantly lower mean HDL particle size than participants with eGFR > 60 mL/min/1.73 m^2^, and larger HDL particles were associated with lower CVD risk [15]. Stefanovic et al. found that in end-stage renal disease patients, the mean HDL size was lower on average by 10% compared to age- and sex-matched post-transplantation subjects [16]. Conversely, using GGE, Calabresi et al. observed no significant differences in HDL-2 and HDL-3 particle sizes between CKD and non-CKD subjects [17]. A modest reduction in HDL-3 particle diameter was observed only in patients undergoing hemodialysis [17].

The differences in the results of the studies may be due to differences in the particle separation methods applied. A study comparing the sizes of lipoproteins separated by GGE and 2D-PAGGE showed that α1-HDL particles covered most HDL-2 subpopulations, while α2, α3, and pre-β1 covered HDL-3 [18]. Moreover, following ultracentrifugation, the preβ1-HDL and preβ2-HDL particles were found in the fraction with a density above 1.21 g/ml; therefore, this fraction may not be visible in the GGE after the initial separation of particles with ultracentrifugation [19]. The researchers also observed that the relationships between NMR-measured HDL size and CVD risk were slightly higher than those identified with GGE, indicating that changes in HDL following ultracentrifugation may affect particle size determination [20]. In our study, we used the 2D-PAGGE method and observed the differences in the mean sizes of α- and preβ2-mobility HDL particles between CKD and non-CKD subjects, which are consistent with the results of NMR studies [15, 16]. Moreover, apart from decreases in the mean sizes of α- and preβ2-HDL particles linked with eGFR values, we observed that individual HDL subpopulations differed in their degrees of size reduction.

Considering the classic HDL maturation model based on the mutual transformation of HDL subpopulation particles that covers gradual particle size enlargement from very small discoidal preβ1-HDL particles, via spherical α4, α3, and α2 particles of increasing sizes, to very large α1-HDL particles [18], we can assume that the disturbed HDL subpopulation particle size profiles in CKD observed by us are related to alterations in the activity or levels of enzymes involved in HDL transformation. Moreover, this suggests that some HDL metabolism stages can be more prone to disturbances related to kidney failure. Our results also confirm that changes in HDL features already occur in the early stages of CKD and worsen with the deterioration of renal function. In CKD, the activity and concentration of LCAT both decrease, leading to decreased remodeling of small discoidal HDL particles into larger spherical particles [17, 21]. Reduced HDL size may also relate to an increased fractional catabolic rate of apoAI as well as oxidative changes of apoAI occurring in CKD, as oxidized apoAI is not only a weaker activator of ABCA1 and LCAT but also has a reduced ability to switch between lipid-free and HDL-bound forms [22–24]. On the other hand, there are also data suggesting that different HDL size-defined subpopulations are secreted into plasma and circulated mainly at the secreted size until they are removed from circulation [14]. Thus, it cannot be excluded that in CKD, the generation of HDL is disturbed, leading to the occurrence of HDL subpopulations of smaller sizes.

Our study is one of the few to examine changes in the sizes of HDL particles in CKD patients. The novelty of our study is that it also assessed the size of eight individual HDL subpopulations in patients in moderate to severe stages of CKD. To the best of our knowledge, this is the first study to report a significant decrease in preβ2-HDL particle size and differences in size reduction degrees for individual HDL subpopulations in CKD. Moreover, we have observed that the electrophoretic mobility of preβ2-HDL relative to α-HDL mobility was significantly higher in CKD patients, which can also confirm a significant impact of CKD on HDL properties that occurs already in moderate stages of kidney dysfunction.

The increased electrophoretic mobility of lipoproteins on agarose gel may be due to their smaller sizes, but it can also be related to the post-translational modification of lipoproteins, such as carbamylation, which affects the surface charge of particles. Carbamylation is a non-enzymatic post-translational protein modification that occurs when a *carbamoyl* moiety (-CONH2) is added to the functional groups of amino acids. It is induced mainly by exposure to the urea dissociation product, cyanate, present in high levels in patients with kidney dysfunction. Additional mediators of protein carbamylation include inflammation, diet, and smoking [25]. Carbamylation affects HDL particles and their antiatherogenic properties. It has been found to reduce HDL ability to activate LCAT, a key enzyme participating in HDL maturation [26], which can be linked with a decreased size of HDL observed in CKD.

Our study also had some limitations. The number of subjects was relatively small, which was related to the rigorous exclusion criteria chosen and the complex HDL separation method applied. The 2D-PAGGE method we used is a very high-resolution method, but it requires a specialized laboratory and is very time-consuming and labor-intensive. Another limitation of this method is that precast gels are not commercially available, and the quality of lipoprotein separation can be affected by differences between individual gels [6]. Moreover, although the measurable signal for each α-mobility HDL subpopulation was obtained for all the patients in our study, we did not observe measurable signals of preβ1-HDL and/or individual preβ2-HDL subpopulations for some samples. This could be related to low levels of HDL subpopulations, inadequate sensitivity of the immunodetection method used, or changes in the conformation of apoAI, resulting in a loss of the specific epitope recognized by the applied monoclonal antibody [27]. Moreover, some patients were taking low-dose statins, that were previously found to alter the size of α-mobility HDL subpopulations when applied in high doses, especially rosuvastatin [28]. However, in our study, none of the patients were on a high dose of statin, and only two patients received rosuvastatin (one patient at a dose of 10 mg/d and one patient at a dose of 20 mg/d). Atorvastatin was administered to nine patients (three, five, and one patient received doses of 10 mg/d, 20 mg/d, and 40 mg/d, respectively). Simvastatin, at doses of 10 mg/d and 20 mg/d, was taken by two and four patients, respectively. Moreover, considering the possible impact of statins on HDL size or mobility, we included statin treatment as a potential covariate in multivariate stepwise linear regression analyses to identify the associations between eGFR CKD-EPI, and HDL particle sizes and mobility. The analysis showed that after adjusting for covariates, the relationships remained statistically significant. Thus, despite the limitations, we can conclude that our results clearly showed that CKD affected HDL size and mobility, and this effect depended on the severity of renal failure and the type of HDL subpopulation.

The metabolic basis for reduced HDL particle size and/or its increased mobility is unknown in both general and CKD populations, and further studies are needed [2]. An important issue is whether and how changes in HDL composition and properties relate to HDL dysfunction in CKD patients. Inflammation, oxidative stress, and carbamylation can affect HDL composition and functionality at different stages of their metabolism, impairing their protective activities [4]. It has been shown that HDL from CKD patients is defective in promoting RCT [29]. CKD also impairs HDL-associated paraoxonase (PON1) activity and the antioxidative capacity of HDL, and it is related to the loss of HDL anti-inflammatory and endothelial protective activities [30]. In our previous study, we also found that CKD reduced the positive impact of HDL on very-low density lipoprotein (VLDL) lipolysis efficiency mediated by lipoprotein lipase (LPL) [31]. However, the causal link between changes in individual HDL subpopulation size, mobility, and HDL dysfunction remains to be evaluated. Further studies are needed to determine whether smaller HDL particles are dysfunctional per se and/or whether they are more prone to being modified and becoming dysfunctional. It also remains to be clarified whether HDL size or mobility analysis may help in CVD risk stratification.

In summary, we found that the mean size of HDL particles with α- and preβ2-mobility was decreased in non-dialysis CKD patients with moderate to severe kidney impairment, and the lower the eGFR values, the lower the mean HDL sizes. Moreover, the preβ2-HDL relative electrophoretic mobility was significantly higher in CKD subjects, and the lower the eGFR values, the higher the mobility that was observed. The degrees of particle size reduction differed for individual HDL subpopulations, being more pronounced for the largest preβ2-mobility particles and the lowest α-mobility HDL particles, thus indicating that individual HDL metabolism stages can be more prone to disturbances related to kidney failure. Further studies are needed to clarify the link between HDL subpopulation size and mobility disturbances and between HDL disturbances and CVD risk in CKD patients.
